# 1,8-Diiodo­anthracene

**DOI:** 10.1107/S1600536810035191

**Published:** 2010-09-08

**Authors:** Waka Nakanishi, Shunpei Hitosugi, Anna Piskareva, Hiroyuki Isobe

**Affiliations:** a Department of Chemistry, Tohoku University, Aoba-ku, Sendai 980-8578, Japan

## Abstract

The mol­ecule of the title compound, C_14_H_8_I_2_, an inter­mediate in the synthesis of organic materials, is nearly planar, the maximum deviation from the mean plane being 0.032 (1) Å for the C atoms and 0.082 (2) Å for the I atoms. In the crystal structure, a sandwich–herringbone arrangement of mol­ecules is observed, whereas a columnar π-stacking arrangement has been reported for the chlorinated congener 1,8-dichloro­anthracene. Similar effects of halogen substituents on the modulation of packing arrangements are reported for halogenated aromatic compounds such as tetra­cenes and chrycenes.

## Related literature

For the synthesis, see: Lovell & Joule (1997 ▶); Goichi *et al.* (2005 ▶). For the crystal structure of related 1,8-dichloro­anthracenes, see: Desvergne *et al.*, (1978 ▶); Benites *et al.*, (1996 ▶). For similar halogen effects on the arrangement of aromatic mol­ecules, see: Moon *et al.* (2004 ▶); Isobe *et al.* (2009 ▶). For an example of synthetic utility of the title compound in organic materials, see: Nakanishi *et al.* (2010 ▶).
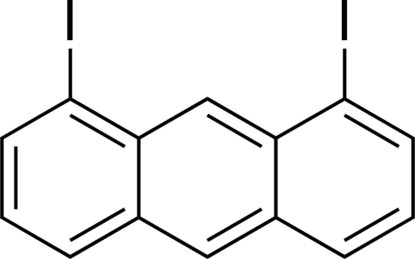


## Experimental

### 

#### Crystal data

C_14_H_8_I_2_
*M* *_r_* = 430.00
Monoclinic, 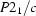

*a* = 10.1167 (11) Å
*b* = 10.8680 (11) Å
*c* = 11.3930 (12) Å
β = 101.829 (1)°
*V* = 1226.0 (2) Å^3^
*Z* = 4
Mo *K*α radiation
μ = 5.10 mm^−1^
*T* = 100 K
0.20 × 0.20 × 0.10 mm

#### Data collection

Bruker APEXII CCD area-detector diffractometer
Absorption correction: multi-scan (*SADABS*; Sheldrick, 1996 ▶) *T*_min_ = 0.429, *T*_max_ = 0.630
13646 measured reflections
2904 independent reflections
2783 reflections with *I* > 2σ(*I*)
*R*_int_ = 0.016

#### Refinement

*R*[*F* ^2^ > 2σ(*F* ^2^)] = 0.014
*wR*(*F* ^2^) = 0.038
*S* = 1.09
2904 reflections
145 parameters
H-atom parameters constrained
Δρ_max_ = 0.62 e Å^−3^
Δρ_min_ = −0.63 e Å^−3^

### 

Data collection: *APEX2* (Bruker, 2006 ▶); cell refinement: *SAINT* (Bruker, 2004 ▶); data reduction: *SAINT*; program(s) used to solve structure: *SHELXS97* (Sheldrick, 2008 ▶); program(s) used to refine structure: *SHELXL97* (Sheldrick, 2008 ▶); molecular graphics: *ORTEP-3* (Farrugia, 1997 ▶) and *Mercury* (Macrae *et al.*, 2008 ▶); software used to prepare material for publication: *SHELXL97*, *Yadokari-XG* (Kabuto *et al.*, 2009 ▶) and *publCIF* (Westrip, 2010 ▶).

## Supplementary Material

Crystal structure: contains datablocks I, global. DOI: 10.1107/S1600536810035191/jh2197sup1.cif

Structure factors: contains datablocks I. DOI: 10.1107/S1600536810035191/jh2197Isup2.hkl

Additional supplementary materials:  crystallographic information; 3D view; checkCIF report

## Figures and Tables

**Fig. 1.**
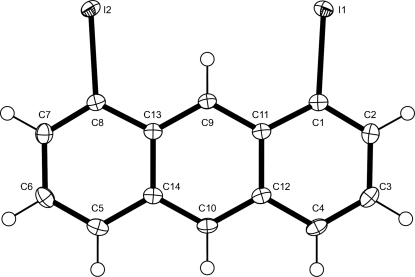
The molecular structure of the title compound with displacement ellipsoids drawn at the 50% probability level.

**Fig. 2.**
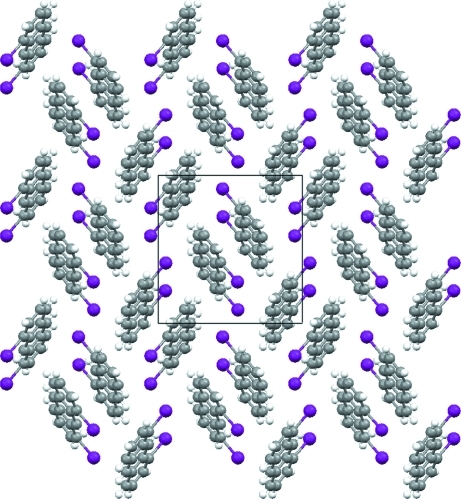
The sandwich-herringbone packing of the title compound, viewed along the a axis.

## supplementary crystallographic information

### Comment

Acenes are important compounds for the development of organic electronics, and the halogenated derivatives are of topical interest due to the unique packing arrangements (Moon *et al.*, 2004; Isobe *et al.*, 2009). The crystal structure of 1,8-dihaloanthracenes has been reported only for a chlorinated compound (Desvergne *et al.*, 1978; Benites *et al.*, 1996), and a columnar π-stacking arrangement of the molecules in the crystal has been revealed. We obtained a single-crystal of 1,8-diiodoanthracene and found a sandwich-herringbone arrangement of the molecules in the crystal. The molecular structure is shown in Fig. 1, and the packing structure is shown in Fig. 2. The distance of π-stacking between the sandwiched dimer is 3.401 Å. The CH-π and halogen-π distances for the herringbone contacts are 2.908 (7) and 3.446 (3) Å, respectively.

### Experimental

The title compound was synthesized from 4,5-diiodo-9-anthrone by a procedure similar to those reported in literatures (Lovell *et al.*, 1997; Goichi *et al.*, 2005). A single-crystal suitable for X-ray crystallographic analysis was obtained by recrystallization from a mixture of hexanes and dichloromethane (5:1).

### Refinement

H atoms were included in calculated positions and treated as riding atoms, with C—H = 0.9 Å (aromatic) and *U*_iso_(H) = 1.2*U*_eq_(C).

### Crystal data

**Table:**

C_14_H_8_I_2_	*F*(000) = 792
*M**_r_* = 430.00	*D*_x_ = 2.330 Mg m^−^^3^
Monoclinic, *P*2_1_/*c*	Mo *K*α radiation, λ = 0.71073 Å
Hall symbol: -P 2ybc	Cell parameters from 5335 reflections
*a* = 10.1167 (11) Å	θ = 2.6–27.8°
*b* = 10.8680 (11) Å	µ = 5.10 mm^−^^1^
*c* = 11.3930 (12) Å	*T* = 100 K
β = 101.829 (1)°	Cubic, green
*V* = 1226.0 (2) Å^3^	0.20 × 0.20 × 0.10 mm
*Z* = 4	

### Data collection

**Table:**

Bruker APEXII CCD area-detector diffractometer	2904 independent reflections
Radiation source: Bruker TXS fine-focus rotating anode	2783 reflections with *I* > 2σ(*I*)
Bruker Helios multilayer confocal mirror	*R*_int_ = 0.016
Detector resolution: 8.333 pixels mm^-1^	θ_max_ = 27.9°, θ_min_ = 2.1°
φ and ω scans	*h* = −13→12
Absorption correction: multi-scan (*SADABS*; Sheldrick, 1996)	*k* = −14→14
*T*_min_ = 0.429, *T*_max_ = 0.630	*l* = −14→14
13646 measured reflections	

### Refinement

**Table:**

Refinement on *F*^2^	Primary atom site location: structure-invariant direct methods
Least-squares matrix: full	Secondary atom site location: difference Fourier map
*R*[*F*^2^ > 2σ(*F*^2^)] = 0.014	Hydrogen site location: inferred from neighbouring sites
*wR*(*F*^2^) = 0.038	H-atom parameters constrained
*S* = 1.09	*w* = 1/[σ^2^(*F*_o_^2^) + (0.0197*P*)^2^ + 0.8912*P*] where *P* = (*F*_o_^2^ + 2*F*_c_^2^)/3
2904 reflections	(Δ/σ)_max_ = 0.005
145 parameters	Δρ_max_ = 0.62 e Å^−^^3^
0 restraints	Δρ_min_ = −0.63 e Å^−^^3^

### Special details

**Table:**

Geometry. All e.s.d.'s (except the e.s.d. in the dihedral angle between two l.s. planes) are estimated using the full covariance matrix. The cell e.s.d.'s are taken into account individually in the estimation of e.s.d.'s in distances, angles and torsion angles; correlations between e.s.d.'s in cell parameters are only used when they are defined by crystal symmetry. An approximate (isotropic) treatment of cell e.s.d.'s is used for estimating e.s.d.'s involving l.s. planes.
Refinement. Refinement of *F*^2^ against ALL reflections. The weighted *R*-factor *wR* and goodness of fit *S* are based on *F*^2^, conventional *R*-factors *R* are based on *F*, with *F* set to zero for negative *F*^2^. The threshold expression of *F*^2^ > σ(*F*^2^) is used only for calculating *R*-factors(gt) *etc*. and is not relevant to the choice of reflections for refinement. *R*-factors based on *F*^2^ are statistically about twice as large as those based on *F*, and *R*- factors based on ALL data will be even larger.

### Fractional atomic coordinates and isotropic or equivalent isotropic displacement parameters (Å^2^)

**Table:**

	*x*	*y*	*z*	*U*_iso_*/*U*_eq_	
I1	0.760513 (11)	1.050372 (9)	0.779962 (10)	0.01704 (4)	
I2	0.242248 (11)	1.052601 (9)	0.591438 (9)	0.01734 (4)	
C8	0.21957 (18)	0.93209 (15)	0.73076 (15)	0.0147 (3)	
C9	0.46782 (17)	0.93129 (14)	0.81821 (14)	0.0138 (3)	
H7	0.4839	0.9827	0.7552	0.017*	
C13	0.33549 (17)	0.89456 (14)	0.81903 (14)	0.0136 (3)	
C7	0.09231 (17)	0.89426 (16)	0.73555 (15)	0.0178 (3)	
H6	0.0179	0.9195	0.6752	0.021*	
C5	0.17608 (18)	0.77913 (15)	0.91729 (15)	0.0174 (3)	
H4	0.1600	0.7278	0.9804	0.021*	
C6	0.07051 (17)	0.81682 (16)	0.83110 (15)	0.0188 (3)	
H5	−0.0185	0.7915	0.8344	0.023*	
C14	0.31114 (17)	0.81568 (14)	0.91435 (14)	0.0144 (3)	
C11	0.57708 (17)	0.89446 (14)	0.90752 (14)	0.0137 (3)	
C12	0.55249 (17)	0.81532 (14)	1.00247 (14)	0.0146 (3)	
C10	0.42066 (17)	0.77828 (15)	1.00274 (14)	0.0158 (3)	
H8	0.4048	0.7258	1.0651	0.019*	
C4	0.66292 (18)	0.77839 (15)	1.09544 (15)	0.0173 (3)	
H3	0.6470	0.7262	1.1580	0.021*	
C1	0.71420 (18)	0.93258 (15)	0.91241 (15)	0.0143 (3)	
C2	0.81750 (17)	0.89582 (15)	1.00237 (15)	0.0171 (3)	
H1	0.9071	0.9225	1.0032	0.020*	
C3	0.79074 (18)	0.81744 (16)	1.09492 (15)	0.0186 (3)	
H2	0.8631	0.7920	1.1572	0.022*	

### Atomic displacement parameters (Å^2^)

**Table:**

	*U*^11^	*U*^22^	*U*^33^	*U*^12^	*U*^13^	*U*^23^
I1	0.01809 (7)	0.01676 (6)	0.01754 (7)	−0.00121 (4)	0.00662 (5)	0.00173 (4)
I2	0.02054 (7)	0.01650 (7)	0.01391 (6)	0.00081 (4)	0.00105 (5)	0.00270 (3)
C8	0.0183 (8)	0.0134 (7)	0.0128 (7)	0.0009 (6)	0.0040 (6)	−0.0012 (5)
C9	0.0178 (7)	0.0117 (7)	0.0126 (7)	0.0006 (6)	0.0042 (6)	0.0000 (5)
C13	0.0178 (8)	0.0097 (7)	0.0139 (7)	0.0001 (6)	0.0045 (6)	−0.0019 (5)
C7	0.0160 (8)	0.0192 (8)	0.0173 (8)	0.0001 (6)	0.0015 (6)	−0.0039 (6)
C5	0.0231 (8)	0.0144 (7)	0.0163 (7)	−0.0032 (6)	0.0083 (6)	−0.0024 (6)
C6	0.0170 (8)	0.0199 (8)	0.0209 (8)	−0.0043 (6)	0.0069 (6)	−0.0057 (6)
C14	0.0193 (8)	0.0105 (7)	0.0141 (7)	−0.0005 (6)	0.0051 (6)	−0.0023 (6)
C11	0.0182 (8)	0.0100 (7)	0.0134 (7)	0.0015 (6)	0.0048 (6)	−0.0012 (5)
C12	0.0200 (8)	0.0104 (7)	0.0135 (7)	0.0015 (6)	0.0040 (6)	−0.0008 (6)
C10	0.0225 (8)	0.0116 (7)	0.0145 (7)	0.0006 (6)	0.0063 (6)	0.0012 (6)
C4	0.0245 (8)	0.0130 (7)	0.0141 (7)	0.0045 (6)	0.0032 (6)	0.0018 (6)
C1	0.0182 (8)	0.0114 (7)	0.0145 (7)	0.0022 (6)	0.0060 (6)	−0.0017 (5)
C2	0.0162 (8)	0.0168 (8)	0.0179 (8)	0.0019 (6)	0.0026 (6)	−0.0030 (6)
C3	0.0218 (8)	0.0170 (8)	0.0152 (7)	0.0052 (6)	−0.0006 (6)	−0.0009 (6)

### Geometric parameters (Å, °)

**Table:**

I1—C1	2.1037 (17)	C6—H5	0.9500
I2—C8	2.1064 (17)	C14—C10	1.396 (2)
C8—C7	1.363 (2)	C11—C1	1.438 (2)
C8—C13	1.439 (2)	C11—C12	1.443 (2)
C9—C11	1.399 (2)	C12—C10	1.394 (2)
C9—C13	1.399 (2)	C12—C4	1.430 (2)
C9—H7	0.9500	C10—H8	0.9500
C13—C14	1.444 (2)	C4—C3	1.362 (3)
C7—C6	1.428 (2)	C4—H3	0.9500
C7—H6	0.9500	C1—C2	1.365 (2)
C5—C6	1.357 (3)	C2—C3	1.424 (2)
C5—C14	1.430 (2)	C2—H1	0.9500
C5—H4	0.9500	C3—H2	0.9500
			
C7—C8—C13	121.86 (16)	C9—C11—C1	123.92 (15)
C7—C8—I2	117.86 (12)	C9—C11—C12	118.95 (15)
C13—C8—I2	120.26 (12)	C1—C11—C12	117.12 (15)
C11—C9—C13	121.88 (15)	C10—C12—C4	121.32 (15)
C11—C9—H7	119.1	C10—C12—C11	119.07 (15)
C13—C9—H7	119.1	C4—C12—C11	119.60 (15)
C9—C13—C8	123.96 (15)	C12—C10—C14	122.18 (15)
C9—C13—C14	119.05 (15)	C12—C10—H8	118.9
C8—C13—C14	116.99 (15)	C14—C10—H8	118.9
C8—C7—C6	120.19 (16)	C3—C4—C12	120.49 (15)
C8—C7—H6	119.9	C3—C4—H3	119.8
C6—C7—H6	119.9	C12—C4—H3	119.8
C6—C5—C14	120.88 (15)	C2—C1—C11	121.93 (15)
C6—C5—H4	119.6	C2—C1—I1	117.90 (13)
C14—C5—H4	119.6	C11—C1—I1	120.17 (12)
C5—C6—C7	120.50 (16)	C1—C2—C3	119.90 (16)
C5—C6—H5	119.8	C1—C2—H1	120.0
C7—C6—H5	119.8	C3—C2—H1	120.0
C10—C14—C5	121.54 (15)	C4—C3—C2	120.95 (16)
C10—C14—C13	118.87 (15)	C4—C3—H2	119.5
C5—C14—C13	119.59 (15)	C2—C3—H2	119.5
			
C11—C9—C13—C8	178.61 (15)	C9—C11—C12—C10	−0.2 (2)
C11—C9—C13—C14	−0.4 (2)	C1—C11—C12—C10	178.63 (14)
C7—C8—C13—C9	−179.73 (16)	C9—C11—C12—C4	−179.06 (15)
I2—C8—C13—C9	−1.4 (2)	C1—C11—C12—C4	−0.2 (2)
C7—C8—C13—C14	−0.7 (2)	C4—C12—C10—C14	178.48 (15)
I2—C8—C13—C14	177.65 (11)	C11—C12—C10—C14	−0.3 (2)
C13—C8—C7—C6	1.1 (2)	C5—C14—C10—C12	−178.59 (15)
I2—C8—C7—C6	−177.28 (12)	C13—C14—C10—C12	0.5 (2)
C14—C5—C6—C7	−0.1 (3)	C10—C12—C4—C3	−178.73 (16)
C8—C7—C6—C5	−0.7 (3)	C11—C12—C4—C3	0.1 (2)
C6—C5—C14—C10	179.61 (16)	C9—C11—C1—C2	178.97 (16)
C6—C5—C14—C13	0.5 (2)	C12—C11—C1—C2	0.2 (2)
C9—C13—C14—C10	−0.1 (2)	C9—C11—C1—I1	−0.2 (2)
C8—C13—C14—C10	−179.24 (14)	C12—C11—C1—I1	−178.97 (11)
C9—C13—C14—C5	178.98 (15)	C11—C1—C2—C3	0.0 (2)
C8—C13—C14—C5	−0.1 (2)	I1—C1—C2—C3	179.15 (12)
C13—C9—C11—C1	−178.17 (15)	C12—C4—C3—C2	0.1 (2)
C13—C9—C11—C12	0.6 (2)	C1—C2—C3—C4	−0.1 (2)
